# Partially Expelled Necrotic Uterine Fibroid After Relugolix Combination Therapy: Case Report and Literature Overview on Adverse Effects of GnRH Antagonists

**DOI:** 10.1155/crog/1926623

**Published:** 2026-03-16

**Authors:** Laura Vona, Anna Pitsillidi, Guenter Noé, Stefano Bettocchi

**Affiliations:** ^1^ Department of Medical and Surgical Sciences, Institute of Obstetrics and Gynaecology, University of Foggia, Foggia, Italy, unifg.it; ^2^ Department of Obstetrics and Gynaecology, Rheinland Klinikum Neuss, Neuss, Germany, unicamp.br; ^3^ Department of OB/GYN, University of Witten Herdecke, Witten, Germany

**Keywords:** adverse effect, estradiol, fibroid, gonadotropin-releasing hormone antagonist, norethisterone acetate, Relugolix

## Abstract

**Background and Clinical Significance:**

Relugolix combination therapy (Relugolix CT) has emerged as an effective oral medical treatment for symptomatic uterine fibroids, offering an alternative to surgical interventions. While generally well tolerated, reports of adverse events beyond the common side effects are limited.

**Case Presentation:**

A 34‐year‐old nulliparous woman presented with abdominal pain and abnormal uterine bleeding. She had previously been diagnosed with a large FIGO Type 1–5 anterior wall fibroid and started on Relugolix CT as a bridge to surgery. At referral, speculum examination revealed a necrotic, malodorous, partially expelled mass protruding through the cervix. Surgical removal under spinal anaesthesia was performed, followed by resection of the intracavitary component. Histopathology confirmed leiomyoma with extensive necrosis. Postoperative imaging showed a residual fibroid, leading to discontinuation of Relugolix and initiation of leuprorelin acetate. The patient reported symptom resolution but was lost to follow‐up after 3 months. This case highlights potential serious but under‐recognized adverse effects associated with Relugolix CT, particularly in patients with large or intracavitary fibroids. Clinicians should maintain vigilance and ensure appropriate monitoring during treatment.

**Conclusion:**

Relugolix CT represents a promising option in uterine fibroid management, but individualized patient evaluation and awareness of possible complications are essential to optimize safety and outcomes. The potential of this therapeutic approach warrants further investigation through randomized clinical trials, while real‐world patient data are equally crucial to strengthen the evidence base and support broader clinical applicability.

## 1. Introduction and Clinical Significance

Uterine fibroids are a common type of benign tumour arising in the uterus, and their development is influenced by ovarian hormones—particularly oestrogen and progesterone—making them the most common tumours of the female reproductive system in women of childbearing age [1]. Uterine fibroids are estimated to affect more than 70% of women during their lifetime. While the majority remain asymptomatic, approximately 25% experience clinically significant symptoms that necessitate medical intervention [2–4]. Although hysterectomy remains the only definitive treatment for uterine fibroids, several uterus‐sparing options are now available for women who wish to preserve fertility. These include myomectomy (via hysteroscopic, laparoscopic or open abdominal approaches), interventional radiology procedures such as uterine artery embolization, magnetic resonance‐guided focused ultrasound and radiofrequency ablation, as well as endometrial ablation [5, 6]. As oestrogen and progesterone are key drivers of fibroid growth, pharmacological management of symptomatic uterine fibroids focuses on modulating these hormonal pathways [7, 8].

GnRH analogues are classified into two groups based on their effects on the pituitary gland: GnRH antagonists and GnRH agonists [9]. GnRH agonists initially induce a transient stimulation of the pituitary gland—referred to as the ‘flare up effect’—which is subsequently followed by downregulation and internalization of GnRH receptors, leading to suppression of the hypothalamic‐pituitary‐gonadal axis [10]. Most side effects experienced during treatment with GnRH agonists stem from oestrogen deficiency and include vasomotor symptoms, reduced libido, sexual dysfunction, vaginal atrophy, osteoporosis and infertility [11]. Newer GnRH antagonists, such as Relugolix and Linzagolix, are nonpeptide, orally administered compounds that competitively and reversibly bind to GnRH receptors in the anterior pituitary. By blocking these receptors, they quickly inhibit the release of follicle‐stimulating hormone (FSH) and luteinizing hormone (LH). Unlike GnRH agonists, these antagonists provide rapid suppression of gonadotropin secretion by directly competing at the receptor level—thereby avoiding the initial surge in hormone levels [12, 13]. To mitigate side effects associated with low oestrogen levels, add‐back therapy is frequently administered alongside GnRH antagonists to maintain systemic oestradiol within a therapeutic range, thereby effectively managing fibroid symptoms while minimizing hypogonadal effects [14]. The most observed adverse reactions associated with this therapy comprise uterine bleeding, headache, diminished libido, vulvovaginal dryness, hot flushes, hyperhidrosis, nausea, alopecia, arthralgia, dizziness and a decrease in bone mineral density. Rare but serious adverse events (AEs) include uterine myoma expulsion, menorrhagia, cholecystitis and pelvic pain [15]. While its efficacy and safety profile have been evaluated in pivotal clinical trials, real‐world data on its adverse effects—particularly less common or delayed‐onset events—remain limited due to its recent introduction to clinical practice. This work presents a clinical case, complemented by a targeted literature review to contextualize current knowledge on the side effect profile of Relugolix combination therapy (Relugolix CT). The case illustrates a rare and clinically significant adverse reaction, while the reviewed evidence summarizes both commonly reported and less frequent treatment‐related events. Together, these elements aim to support clinicians in recognizing potential complications and optimizing patient management.

Unlike most previously reported cases, which predominantly involved pedunculated submucosal fibroids (FIGO Type 0) treated with Relugolix monotherapy, the present case describes a large FIGO Type 1–5 fibroid undergoing extensive necrosis and partial expulsion after only 30 days of Relugolix CT used as a bridge to surgery. This clinical scenario expands current knowledge on the spectrum of fibroid behaviour under GnRH antagonist treatment and underscores the need for careful monitoring, particularly in patients with large or partially intracavitary leiomyomas.

## 2. Case Presentation

A 34‐year‐old Caucasian nulliparous woman was referred to our institution in October 2024 with abdominal pain and abnormal uterine bleeding. She had previously been hospitalized in August 2024 for heavy menstrual bleeding, pelvic pain and anaemia (haemoglobin 8 g/dL), at which time a large FIGO Type 1–5 anterior wall uterine fibroid measuring 89 × 75 × 69 mm was diagnosed. Relugolix CT was initiated as a bridge to surgery, and the patient had been adherent to treatment for approximately 30 days prior to referral. Upon re‐evaluation, speculum examination revealed a necrotic, malodorous, partially expelled mass measuring approximately 40 × 25 mm, occupying the cervical canal and partially protruding into the vagina. Despite the presence of malodorous necrotic tissue, the patient was afebrile, inflammatory markers were within normal limits and there were no clinical signs of pelvic infection; therefore, antibiotic therapy was not required. Laboratory tests showed a haemoglobin level of 9.5 g/dL.

Surgical removal was performed under spinal anaesthesia, beginning with extraction of the visible portion using ring forceps, followed by resection of the intracavitary component using a 26 Fr resectoscope (Karl Storz, Tuttlingen, Germany). Cervical dilation was not required due to spontaneous dilatation from the prolapsing fibroid.

Postoperative transvaginal ultrasound revealed a residual mass measuring 61 × 45 × 50 mm. Histopathological analysis confirmed the presence of leiomyomatous tissue with extensive areas of necrosis. Considering these findings, treatment with Relugolix CT was discontinued, and the patient was initiated on leuprorelin acetate 11.75 mg. Follow‐up imaging and hysteroscopic evaluation were scheduled for 3 months later. At the 30‐day follow‐up, the patient reported no further episodes of vaginal bleeding. After 3 months of leuprorelin therapy, however, the patient declined further follow‐up.

## 3. Discussion

### 3.1. Drug Properties

**FIGURE 1:**
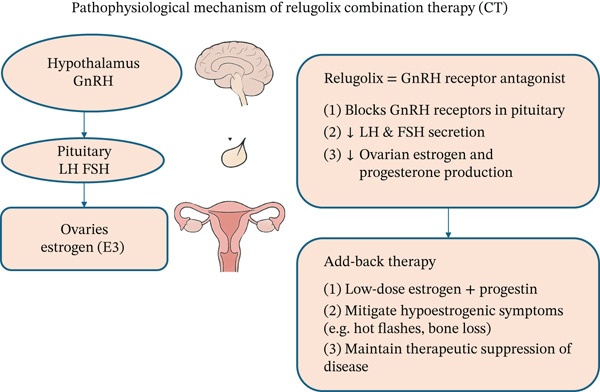
Pathophysiological mechanism of Relugolix CT.

Relugolix is an orally administered, nonpeptide antagonist of GnRH receptors. It acts by competitively binding to GnRH receptors in the anterior pituitary, thereby inhibiting their activation. Reduced FSH levels inhibit follicular maturation, consequently decreasing ovarian oestrogen production [13]. Relugolix suppresses the LH surge and reduces progesterone and estradiol levels, improving fibroid‐related symptoms such as heavy menstrual bleeding [16]. Exogenous E2, identical to endogenous estradiol, relieves hypoestrogenic symptoms and supports bone health [17]. Norethisterone acetate counteracts the endometrial hyperplasia risk of unopposed oestrogen by promoting endometrial differentiation [18] (Figure 1).

With the fixed‐dose Relugolix/E2/norethisterone acetate (40/1/0.5 mg), estradiol levels reflect both Relugolix‐induced suppression and 1‐mg exogenous E2, stabilizing in the early follicular range (20–50 pg/mL), which helps limit bone loss and vasomotor symptoms and supports long‐term treatment of uterine fibroids [19]. Oral GnRH antagonists offer rapid, dose‐dependent oestrogen suppression and quick reversibility [20], but a rebound effect may occur, with 79% of women resuming menses within 2 months—often with heavy first bleeding due to abrupt hormonal reactivation [21, 22].

### 3.2. Landmark Trials and Adverse Effects

Myfembree is approved for heavy menstrual bleeding from uterine fibroids for up to 24 months, whereas Ryeqo is indicated for moderate–severe fibroid symptoms without a defined treatment limit [14]. In two Phase 3 trials, both Relugolix CT and monotherapy significantly reduced menstrual bleeding versus placebo (*p* < 0.001) [23] and improved key secondary outcomes, including amenorrhea rates, menstrual blood loss, pelvic pain, anaemia and uterine volume reduction (~14%), although fibroid volume reduction was not statistically significant 14, 24]. Treatment discontinuation due to AEs occurred in 3.9% of patients on Relugolix CT and 4.3% on placebo, with uterine bleeding as the most common cause (1.2%) [24]. Relugolix CT did not produce clinically relevant BMD loss [15]. The most frequent AEs (≥ 3% and higher than placebo) were hot flushes/hyperhidrosis (10.6% vs. 6.6%), abnormal uterine bleeding (6.3% vs. 1.2%), alopecia (3.5% vs. 0.8%), decreased libido (3.1% vs. 0.4%) and hypertension (7.0% vs. 0.8%) [24]. Less common events included irritability, dyspepsia and breast cysts [25], with no liver injury, lipid abnormalities or endometrial pathology observed [26].

Eight SAEs occurred in the Relugolix CT group, including fractures, hematemesis, hypothyroidism, pelvic pain and fibroid expulsion; five SAEs were reported in the delayed‐CT group and six in placebo [14, 24]. In the 52‐week extension, one haemorrhage occurred in the continuous CT group; additional SAEs in other groups included fractures, cholecystitis, leiomyoma progression, atrial fibrillation and disc protrusion [14]. Over 104 weeks, BMD change was minimal (0.04%) among long‐term users, and low‐trauma fractures were rare (0.6% of 634 patients) [27, 28]. No new safety signals emerged, SAEs remained uncommon (0.09%) and no deaths occurred [23]. Overall, SAEs were mainly unrelated to treatment [14]. Table 1 summarizes the principal adverse effects.

**TABLE 1:** **Adverse effect**s.

Category	Adverse events	Frequency
Common (≥ 5%)	Headache, hot flushes, night sweats, nausea	Generally mild to moderate; typically resolve over time
Gynaecologic	Fibroid expulsion, irregular uterine bleeding, spotting	Expulsion more likely with submucosal fibroids; bleeding may occur early in therapy
Vascular	Venous thromboembolism (VTE)	Rare; risk elevated in predisposed patients (consider contraindications)
Psychic sphere	Depression, mood swings, irritability	Monitor in patients with psychiatric history
Skeletal health	Decrease in bone mineral density (BMD)	Minimal with add‐back therapy; assess BMD if long‐term use or at‐risk
Others	Fatigue, abdominal pain, back pain, breast tenderness	Generally nonsevere and transient

To further investigate the causes that may have led to this rare complication observed in our case report, we conducted a careful analysis of the literature on the occurrence of fibroid prolapse and/or expulsion following treatment with GnRH analogues. Several studies have reported this phenomenon, although the exact pathophysiological mechanisms remain unclear.

In Phase 3 placebo‐controlled clinical trials, uterine fibroid prolapse and uterine fibroid expulsion were reported in women treated with Relugolix, oestradiol and norethindrone acetate [29]. Indeed, women with known or suspected submucosal uterine fibroids who initiate treatment with Relugolix CT should be informed about the possibility of uterine fibroid prolapse or expulsion, as per EMA guidance [26].

Muzii et al. enrolled 36 patients in a study investigating the preoperative use of Relugolix CT for abnormal uterine bleeding (AUB) associated with uterine fibroids. One patient did not complete the study and required emergency surgical intervention after 2 months of medical therapy due to the expulsion of a submucosal fibroid, which had decreased in volume during treatment [30].

Ishizawa et al. reported the first documented case of massive vaginal bleeding during treatment with Relugolix monotherapy, attributed to the prolapse of a submucosal fibroid (FIGO Type 0, pedunculated). A 55‐year‐old woman with regular menstrual cycles but recurrent atypical vaginal bleeding was diagnosed with a 67 × 51 × 51 mm submucosal fibroid, which had descended into the cervical canal, as confirmed by transvaginal ultrasound (TV‐US) and magnetic resonance imaging (MRI). Relugolix (40 mg daily) was prescribed for symptomatic management. On day 35 of treatment, the patient experienced a sudden onset of massive vaginal bleeding and presented to the emergency department due to leiomyoma prolapse. She underwent an uncomplicated abdominal hysterectomy [31].

Wada et al. retrospectively analysed clinical records of 17 patients treated with Relugolix monotherapy (40 mg daily for up to 6 months) for submucosal fibroids. Two patients, both with FIGO Type 0 fibroids, developed acute severe haemorrhage requiring urgent surgical intervention and blood transfusions. In one case, severe bleeding occurred on day 27 of treatment; pelvic examination and MRI identified a pedunculated fibroid measuring 33 × 27 × 24 mm prolapsing through the cervical canal. In this case, fibroid prolapse was hypothesized to result from a rapid reduction in fibroid volume [32].

The second patient had a pretreatment pedunculated fibroid measuring 61 × 43 × 31 mm prolapsing through the cervix. On Day 5 of treatment, she developed heavy bleeding, with pelvic examination revealing haemorrhage originating from the surface of the prolapsed fibroid. Relugolix causes a rapid reduction in oestradiol levels; this hormonal suppression may lead to increased fragility of the fibroid and its peduncle, thereby elevating the risk of haemorrhage. In the second case, bleeding may have been triggered by contact between the friable fibroid surface and the vaginal wall [32].

The reported cases of fibroid prolapse during treatment with GnRH analogues support an association between medical therapy and this rare complication. In all cases, prolapse occurred after a significant reduction in fibroid size, suggesting that rapid volume decrease may be a key mechanism [33–36]. A similar mechanism has been observed in the postpartum period, where fibroid prolapse has also been reported [37].

Furthermore, in cases involving GnRH antagonists, the time from treatment initiation to prolapse appears to be shorter than that reported with GnRH agonists. Unlike GnRH agonists, antagonists do not induce an initial flare‐up phase and lead to a more immediate suppression of oestradiol, which may explain the earlier onset of prolapse [16, 38]. These findings suggest that GnRH antagonists, such as Relugolix, may be associated with an earlier onset of fibroid prolapse compared to agonists [31].

In the cases described in the literature, fibroid prolapse has frequently been associated with pedunculated submucosal fibroids (FIGO Type 0). Compared with previously published cases, including the recent report by Ishizawa et al., in which fibroid prolapse occurred in a FIGO Type 0 leiomyoma treated with Relugolix monotherapy, the present case is distinguished by the fibroid′s mixed FIGO 1–5 morphology, the early onset of necrotic degeneration and its occurrence during combination therapy administered as a preoperative strategy. In our patient, the fibroid was of considerable size and exhibited both intramural and intracavitary components. We hypothesize that treatment‐induced fibroid shrinkage may have led to dynamic changes in fibroid localization, resulting in a functional reclassification according to the FIGO system, as previously described in the literature [39, 40]. Although a temporal association between Relugolix CT and fibroid necrosis with partial expulsion was observed, this case does not establish a causal relationship. Other contributing factors, including fibroid size, partial intracavitary extension and ischemic degeneration, may have played a role in the observed clinical course. Therefore, these findings should be interpreted with caution and regarded as hypothesis‐generating rather than confirmatory.

A limitation of this report is the loss of patient follow‐up after 3 months, which precluded assessment of long‐term outcomes, including fertility potential, durability of symptom control and evolution of the residual fibroid. These aspects warrant further investigation in future studies.

## 4. Conclusions

Relugolix CT represents a significant advancement in the medical management of symptomatic uterine fibroids, offering an effective and well‐tolerated oral alternative to traditional therapies. However, emerging clinical experience underscores the need for continued vigilance regarding its safety profile. Although most adverse effects are mild to moderate and well‐characterized—such as hot flushes, headaches, and menstrual changes—clinicians should be aware of less common but potentially serious outcomes, including fibroid degeneration and expulsion. The therapeutic potential of this modality warrants further evaluation through randomized clinical trials, while evidence derived from routine clinical practice is equally important to ensure the robustness and external validity of the conclusions. Individualized treatment planning, patient education and timely follow‐up remain key strategies to optimize outcomes and ensure patient safety.

**Nomenclature**
CT: Combination therapy
GnRH: Gonadotropin‐releasing hormone
FSH: Follicle‐stimulating hormone
LH: Luteinizing hormone
E2: Estradiol
TV‐US: Transvaginal ultrasound
RMI: Magnetic resonance imaging
BMD: Bone mineral density
AUB: Abnormal uterine bleeding

## Author Contributions

Conceptualization, S.B., A.P. and L.V.; investigation, L.V. and A.P.; resources, A.P. and L.V.; methodology, A.P. and L.V.; formal analysis, A.P. and G.N.; data curation, L.V. and S.B.; software, A.P.; validation, S.B. and G.N.; writing/original draft preparation, review and editing, A.P. and L.V.; project administration, G.N.; visualization, G.N. and S.B.; supervision, G.N. and A.P.

## Funding

Open access publishing facilitated by Universita degli Studi di Foggia, as part of the Wiley ‐ CRUI‐CARE agreement.

## Disclosure

All authors have read and agreed to the published version of the manuscript.

## Ethics Statement

The study was conducted in accordance with the Declaration of Helsinki and exempt from the Institutional Review Board of Azienda Ospedaliero—Universitaria Policlinico Riuniti di Foggia (Foggia, Italy) due to the retrospective nature of the case report. All patient data were anonymized, and the patient provided written informed consent for the use of their clinical information and images.

## Consent

Written informed consent was obtained from the patient for publication of this case report.

## Conflicts of Interest

The authors declare no conflicts of interest.

## Data Availability

The data that support the findings of this study are available from the corresponding author upon reasonable request.
